# Extracellular vesicles do not contribute to higher circulating levels of soluble LRP1 in idiopathic dilated cardiomyopathy

**DOI:** 10.1111/jcmm.13211

**Published:** 2017-05-29

**Authors:** Santiago Roura, Carolina Gálvez‐Montón, David de Gonzalo‐Calvo, Ana Gámez Valero, Paloma Gastelurrutia, Elena Revuelta‐López, Cristina Prat‐Vidal, Carolina Soler‐Botija, Aida Llucià‐Valldeperas, Isaac Perea‐Gil, Oriol Iborra‐Egea, Francesc E. Borràs, Josep Lupón, Vicenta Llorente‐Cortés, Antoni Bayes‐Genis

**Affiliations:** ^1^ Heart Failure and Cardiac Regeneration (ICREC) Research Program Health Science Research Institute Germans Trias i Pujol (IGTP) Badalona Spain; ^2^ Center of Regenerative Medicine in Barcelona Barcelona Spain; ^3^ CIBERCV Instituto de Salud Carlos III Madrid Spain; ^4^ Cardiovascular Research Center CSIC‐ICCC IIB‐Sant Pau Hospital de la Santa Creu i Sant Pau Barcelona Spain; ^5^ Innovation in Vesicles and Cells for Application in Therapy Group IGTP Badalona Spain; ^6^ Nephrology Service Germans Trias i Pujol University Hospital (HUGTiP) Badalona Spain; ^7^ Cardiology Service HUGTiP Badalona Spain; ^8^ Department of Medicine Barcelona Autonomous University (UAB) Barcelona Spain; ^9^ Institute of Biomedical Research of Barcelona (IIBB) Spanish National Research Council (CSIC) Barcelona Spain

**Keywords:** biomarker, idiopathic dilated cardiomyopathy, extracellular vesicles, sLRP1, size‐exclusion chromatography

## Abstract

Idiopathic dilated cardiomyopathy (IDCM) is a frequent cause of heart transplantation. Potentially valuable blood markers are being sought, and low‐density lipoprotein receptor‐related protein 1 (LRP1) has been linked to the underlying molecular basis of the disease. This study compared circulating levels of soluble LRP1 (sLRP1) in IDCM patients and healthy controls and elucidated whether sLRP1 is exported out of the myocardium through extracellular vesicles (EVs) to gain a better understanding of the pathogenesis of the disease. LRP1 α chain expression was analysed in samples collected from the left ventricles of explanted hearts using immunohistochemistry. sLRP1 concentrations were determined in platelet‐free plasma by enzyme‐linked immunosorbent assay. Plasma‐derived EVs were extracted by size‐exclusion chromatography (SEC) and characterized by nanoparticle tracking analysis and cryo‐transmission electron microscopy. The distributions of vesicular (CD9, CD81) and myocardial (caveolin‐3) proteins and LRP1 α chain were assessed in SEC fractions by flow cytometry. LRP1 α chain was preferably localized to blood vessels in IDCM compared to control myocardium. Circulating sLRP1 was increased in IDCM patients. CD9‐ and CD81‐positive fractions enriched with membrane vesicles with the expected size and morphology were isolated from both groups. The LRP1 α chain was not present in these SEC fractions, which were also positive for caveolin‐3. The increase in circulating sLRP1 in IDCM patients may be clinically valuable. Although EVs do not contribute to higher sLRP1 levels in IDCM, a comprehensive analysis of EV content would provide further insights into the search for novel blood markers.

## Introduction

Dilated cardiomyopathy (DCM) is the most common cause of non‐ischaemic heart failure leading to heart transplantation 1. In the majority of cases, the primary cause is unknown, giving rise to the term idiopathic dilated cardiomyopathy (IDCM). The most relevant pathological and clinical traits include patchy interstitial fibrosis, degenerated cardiomyocytes, marked cardiac endothelial deficits, ventricular chamber enlargement or dilatation, systolic dysfunction with normal left ventricular (LV) wall thickness, progressive LV contractile dysfunction, ventricular and supraventricular arrhythmias, thromboembolism and sudden death 2, 3, 4, 5.

Biomarkers are indicators of variation in normal and pathological processes that are objectively measurable in biological systems or samples 6. As such they are becoming clinically helpful for assessing survival time, as predictive tools in testing safety, toxicity and therapeutic efficacy, for following disease progression, and to avoid redundant treatments and related toxicities. The search for reliable blood markers is ongoing in IDCM 7. In this context, a variety of biomarkers in distinct categories (myocyte stress and stretch, myocyte apoptosis, cardiac interstitium, inflammation, oxidative stress, cardiac energetics, neurohormones and renal biomarkers) have been linked to heart failure, but only natriuretic peptides have been routinely adopted 8, 9. However, others, including high‐sensitivity troponin T and ST2, are currently being introduced into clinical practice 10, 11, 12.

Low‐density lipoprotein (LDL) receptor‐related protein 1 (LRP1) is a multifunctional receptor of the LDL receptor family that mediates the clearance of a variety of structurally diverse extracellular molecules 13. LRP1 has a soluble form (sLRP1) 14, 15, 16, which can be detected in the systemic circulation and other biofluids 17, 18, 19. sLRP1 is composed of the α chain and a ~55‐kDa fragment of the β chain and is generated by endoproteolysis of the membrane β chain segment 18, 20. Recently, increased circulating sLRP1 levels have been associated with severe hypercholesterolaemia 14 and epicardial adipose tissue 21.

Thus, we sought to determine the myocardial expression and localization of the LRP1 α chain and whether it preferentially binds vessels in IDCM compared to control myocardium. We then compared circulating levels of sLRP1 in plasma samples from IDCM patients and healthy controls. Finally, we explored whether sLRP1 is exported out of the myocardium *via* extracellular vesicles (EVs).

## Materials and methods

### Study population

#### Blood collection and plasma processing

Twenty IDCM patients from a multidisciplinary heart failure clinic and 15 age‐ and sex‐matched healthy controls with no cardiovascular disorders were enrolled for peripheral venous blood extraction. All laboratory measurements were performed by experienced staff blinded to the patients' clinical characteristics. The main demographic and clinical characteristics of the patients are summarized in Table 1. In particular, all patients were selected in an ambulatory setting, and no one received cathecolamine infusion close to the inclusion in the study. IDCM was diagnosed by 2D echocardiography by standard methods. Left ventricular (LV) ejection fraction was calculated by the Simpson method using 2C and 4C views. Coronary angiography was performed in the need for diagnosis of exclusion. Fourteen of the 20 patients had coronary angiography. No coronary lesions were found in 12 of them, and one single vessel disease, not considered causative of the dilated cardiomyopathy, was found in two patients. The other six patients without coronary angiography had absence of coronary symptoms and cardiac SPECT or cardiac RN negative for ischaemic heart disease or null suspicion of associated coronary disease. The duration of heart failure was 68.3 ± 60 months, median 40.6 (Q1–Q3 20.3–113.6). Also, none of the patients in the study underwent heart transplantation along follow‐up, and there were just two women included with ages of 50 and 61 years. The study protocol was approved by the Clinical Research Ethics Committee of our institution and conformed to the principles outlined in the Declaration of Helsinki. Written informed consent was obtained from each participant.

**Table 1:** Demographic and clinical characteristics of idiopathic dilated cardiomyopathy patients

		*n*
Age, years	62.3 ± 11.5	20
Male	18 (90)	20
Hypertension	10 (50)	20
Diabetes mellitus	9 (45)	20
Heart rate, bpm	71.3 ± 9.4	20
Systolic blood pressure, mmHg	123.9 ± 21.4	20
Diastolic blood pressure, mmHg	74.8 ± 11.7	20
Ventricular end‐systolic diameter	60.4 ± 9.3	20
Ventricular end‐diastolic diameter	49.5 ± 7.9	20
Dyslipidemia	14 (70)	20
Total cholesterol, mg/dl	183.4 ± 39.5	20
Triglycerides, mg/dl	145.1 ± 89.2	19
LDL cholesterol, mg/dl	114.5 ± 29.6	15
HDL cholesterol, mg/dl	49.6 ± 18.8	16
LVEF	36.1 ± 11.7	20
NYHA functional class, II/III	14/4 (80/20)	20
Treatment		20
Diuretics	17 (85)	
ACEI/ARB	17 (85)	
Beta‐blocker	18 (90)	
MRA	15 (75)	
Digoxin	2 (10)	
Statins	9 (45)	

Data are presented as mean ± S.D. or *n* (%).

ACEI: angiotensin‐converting enzyme inhibitor; ARB: angiotensin II receptor blocker; HDL: high‐density lipoprotein; LDL: low‐density lipoprotein; LVEF: left ventricular ejection fraction; MRA: mineralocorticoid receptor antagonist; NYHA: New York Heart Association.

Peripheral blood was collected following standard procedures that minimize contamination by platelet‐derived vesicles 22. Isolated platelet‐free plasma samples were frozen at −80°C until used in enzyme‐linked immunosorbent assays (ELISA) and purification of EVs.

#### Autopsy material

Full‐thickness left ventricle (LV) collected from explanted IDCM hearts (*n* = 9) and control hearts from non‐cardiac decedents (*n* = 5) were used for histomorphological analysis 4, 23, 24. The demographic and clinical characteristics of patients were previously summarized in detail 24. In brief, eligible IDCM criteria included non‐ischaemic origin, NYHA class II or higher, LV end‐diastolic diameter ≥70 mm and LV ejection fraction <25%, and neither evidence of myocarditis nor family history of cardiomyopathy were reported. All patients were receiving medical treatment according to the guidelines of the European Society of Cardiology, with diuretics 89 %, angiotensin‐converting enzyme inhibitors 89 %, β‐blockers 45 %, aldosterone antagonists 55 %, digoxin 55 % and statins 45 %, and total cholesterol and triglycerides levels were 136.5 and 60.2 mg/dl, respectively. Non‐diseased hearts were mainly obtained from donors with neurological death caused by traffic accident. The hearts were initially considered for cardiac transplantation but were subsequently deemed unsuitable for transplantation either because of blood type or size incompatibility. All donors had normal LV function and no history of myocardial disease or active infection.

Comparatively, the demographic and clinical characteristics of patients from whom blood and tissue samples were extracted were similar except for the LV ejection fraction which was more depressed (15–23.75%) in the myocardial tissue group as these patients were undergoing cardiac transplantation. All patients were also diagnosed with IDCM by the same criteria.

### Measuring sLRP1 levels

sLRP1 levels were measured using a commercially available ELISA (Uscn Life Science Inc., Houston, TX) according to the manufacturer's instructions. The assay had a within‐ and between‐assay variation coefficient of <10% and 12%, respectively, and a limit of detection of 0.156 ng/ml. The assay showed no remarkable cross‐reactivity or interference between sLRP1 and analogues. Associations between sLRP1 concentration, blood lipid parameters, and clinical variables were then analysed.

### Extracellular vesicle isolation and characterization

Extracellular vesicles isolation by size‐exclusion chromatography (SEC) has been widely reported (Appendix S1) 25, 26, 27, 28, 29, 30. The size distribution and concentration of plasma‐derived EVs were determined by nanoparticle tracking analysis (NTA) (Appendix S1). SEC‐purified fractions were also analysed for the presence of specific vesicular markers (CD9 and CD81) 25, caveolin‐3 and LRP1 α chain by flow cytometry, as described previously (Appendix S1) 28. Finally, SEC fractions with the highest mean fluorescence intensity (MFI) for exosomal markers on a FACS‐based assay were also selected for cryo‐electron microscopy 22.

### Immunohistology and myocardial lipid content analysis

Ten‐millimetre‐thick cryosections of LV samples were fixed with 4% paraformaldehyde (Sigma–Aldrich^®^, St. Louis, MO), permeabilized with 0.2% Triton X‐100 (Sigma–Aldrich^®^) and blocked in 20% horse serum (Invitrogen, Carlsbad, CA) for 1 hr at room temperature. Cryosections were stained using antibodies against human LRP1 α chain (1:50; Invitrogen), cardiac troponin I (cTnI) (1:200; Santa Cruz Biotechnology, Inc., Dallas, TX), von Willebrand Factor (vWF) (1:100; BD Pharmingen, San Diego, CA) and CD31 (1:50; Abcam, Cambridge, UK), and with biotinylated GSLI B4 isolectin (1:50; Griffonia simplicifolia lectin I B4; Vector Labs, Burlingame, CA). Alexa Fluor 488‐conjugated streptavidin and Cy2, Cy3 and Cy5 (1:500; Jackson ImmunoResearch Laboratories, West Grove, PA) secondary antibodies were also used. All sections were counterstained with 4',6‐diamidino‐2‐phenylindole dihydrochloride (DAPI) (1:10 000; Sigma‐Aldrich) and analysed by confocal microscopy (Axio Observer Z1; Zeiss, Oberkochen, DE). Quantitative immunohistochemical measurements were completed with the Image‐Pro Plus software (6.2.1 version; MediaCybernetics, Rockville, MD).

On 4‐μm paraffin slices, Picrosirius red staining was performed to analyse collagen (Col) volume fraction (CVF), and Col type I (red/yellow) and Col type III (green) under a computer‐associated Leica DMI 6000B microscope with a polarized filter.

One aliquot of frozen tissue (10 mg) was homogenized in 0.1 M NaOH for lipid content extraction. Subsequently, the amounts of cholesteryl ester (CE), free cholesterol (FC) and triglyceride (TG) were analysed by thin layer chromatography as described previously (Appendix S1) 23.

### Statistical analysis

Data are presented as mean ± S.D. Kolmogorov–Smirnov analysis was used to check for normality of data. Statistical analyses were performed using the Student's *t*‐test, and the Mann–Whitney test for non‐parametric data, using SPSS 19.0.1 (SPSS, Inc., Chicago, IL). Associations between sLRP1 and blood lipid levels or clinical variables were assessed by Pearson correlation for continuous variables and linear regression for categorical variables. Differences were considered significant when *P* < 0.05.

## Results

### LRP1 α chain is preferentially localized in the myocardial vasculature in IDCM patients

First, to confirm fibrosis as a hallmark of failing hearts, we measured myocardial deposition of collagen (Col) by staining with Picrosirius red (Fig. S1). A significant CVF accumulation was found in IDCM compared to control myocardium (*P* = 0.048). Additionally, increased Col I and Col III amounts were also found in IDCM patients (*P* = 0.045 and *P* = 0.005, respectively).

**Figure 1:**
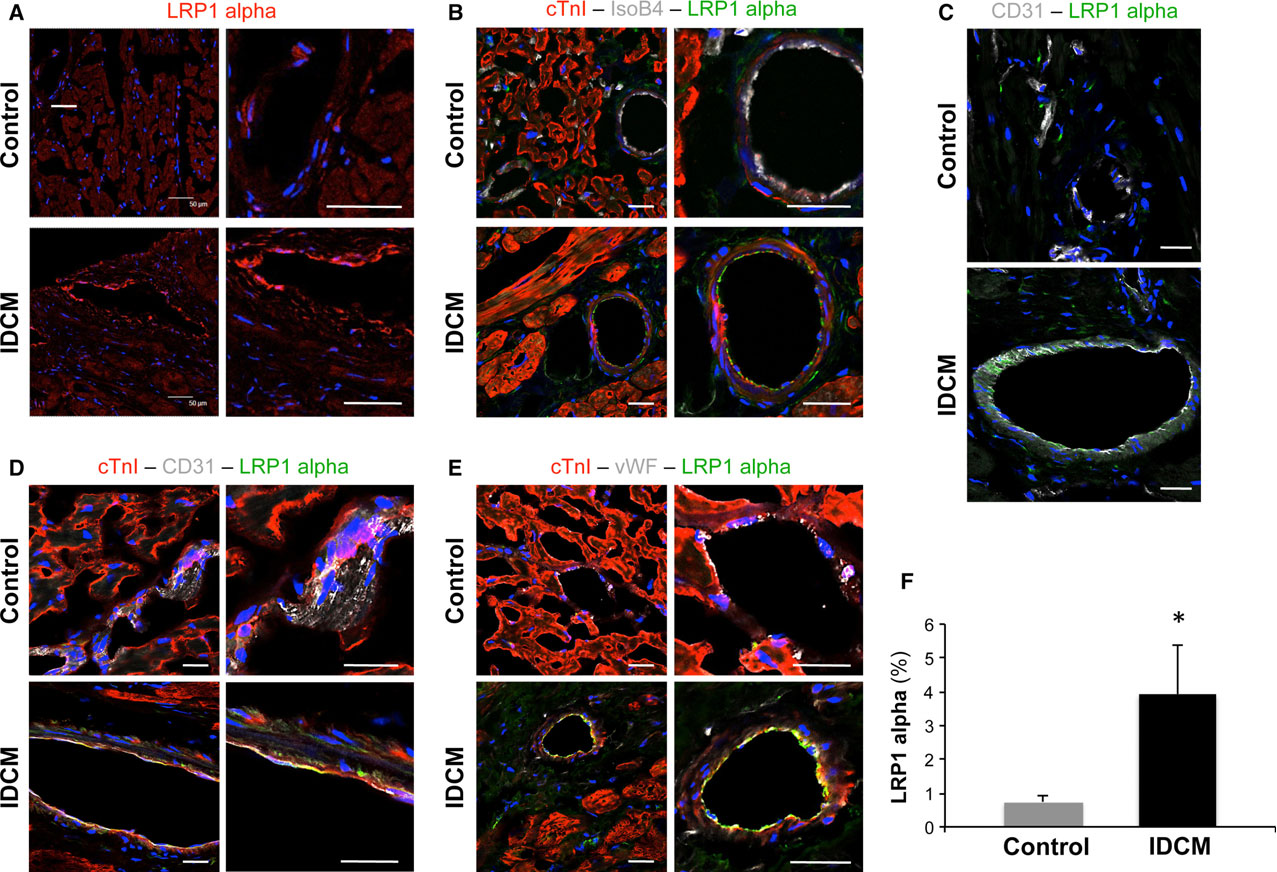
Analysis of myocardial LRP1 α chain expression and localization. Representative confocal microscope images showing specific global detection of LRP1 α chain (red) (**A**) and more detailed localization of LRP1 α chain (green) in IsoB4—(grey) (**B**), CD31—(grey) (**C** and **D**) and vWF—(grey) (**E**) positive vessels. Cardiac muscle and cell nuclei are counterstained using an anti‐cTnI antibody (red) and DAPI (blue), respectively. Scale bars = 50 μm (**F**) Histogram represents quantification of LRP1 α chain positivity as percentage of arbitrary units per area. *P = 0.02.

To explore whether plasma sLRP1 levels are potentially valuable for IDCM, we then assessed myocardial expression of LRP1 α chain using immunohistochemistry. Higher amounts of LRP1 α chain were found in myocardium from IDCM patients compared to controls (*P* = 0.02) (Fig. 1). Remarkably, LRP1 α chain also localized differently between the two groups. In IDCM, LRP1 α chain was mainly found in myocardial vessels positive for isolectin B4, vWF or CD31 and, thus, more exposed to the systemic circulation (Fig. 1).

**Figure 2:**
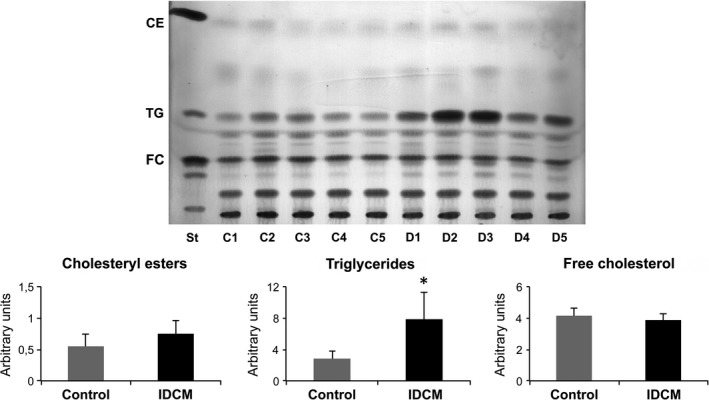
Assessment of myocardial lipid content. Distribution of cholesteryl esters (CE), triglycerides (TG) and free cholesterol (FC) in both groups (St = standard, C = control and D = dilated cardiomyopathy). Histograms represent the quantification of total TG, CE and FC. *n* = 5 each control and idiopathic dilated cardiomyopathy (IDCM). *P = 0.026.

Subsequently, because LRP1 endoproteolysis and secretion has been linked to hypercholesterolaemic conditions, we investigated possible differences in the myocardial lipid content. As shown in Figure 2, although TG content significantly increased in patients compared to controls (*P* = 0.026), no differences in CE or FC amounts were found between the groups (*P* = 0.16 and *P* = 0.35, respectively).

### Circulating sLRP1 levels are higher in IDCM patients than controls

**Figure 3:**
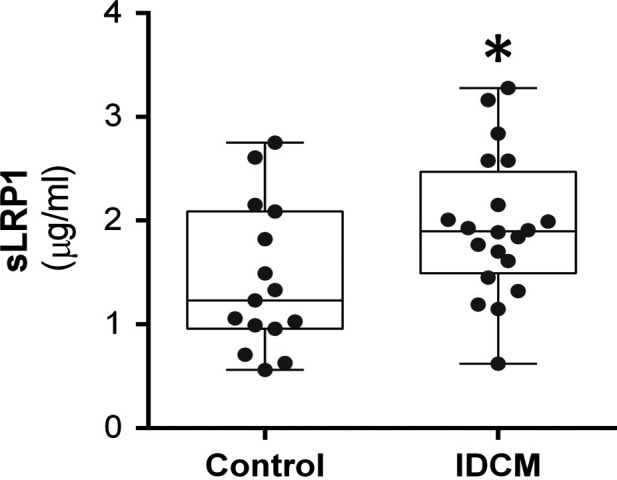
Increased levels of circulating sLRP1 in idiopathic dilated cardiomyopathy (IDCM) patients. The histogram represents quantification of circulating sLRP1 by ELISA. *P = 0.034. *n* = 15 control and *n* = 20 IDCM.

Next, we analysed sLRP1 levels in platelet‐free plasma. As shown in Figure 3, ELISA analyses revealed significantly higher levels of sLRP1 in plasma from IDCM patients compared to controls (*P* = 0.034). To evaluate a possible association or relationship between increased sLRP1 levels and hypercholesterolaemia, we focused on blood lipid parameters. Interestingly, we found no association between sLRP1 levels and any of the lipid parameters, including cholesterol (*r* = 0.04, *P* = 0.87), TGs (*r* = 0.15, *P* = 0.53), HDL (*r* = −0.11, *P* = 0.67) and LDL (*r* = 0.20, *P* = 0.47). Preliminary analyses also revealed no correlation between sLRP1 concentrations and clinical variables, such as LV ejection fraction (*r* = 0.35, *P* = 0.13), age (*r* = 0.004, *P* = 0.99), sex (*P* = 0.37), diabetes mellitus (*P* = 0.27), hypercholesterolaemia (*P* = 0.73), NYHA functional class (*P* = 0.86) and statin treatment (*P* = 0.14).

### LRP1 α chain is not secreted *via* EVs

**Figure 4:**
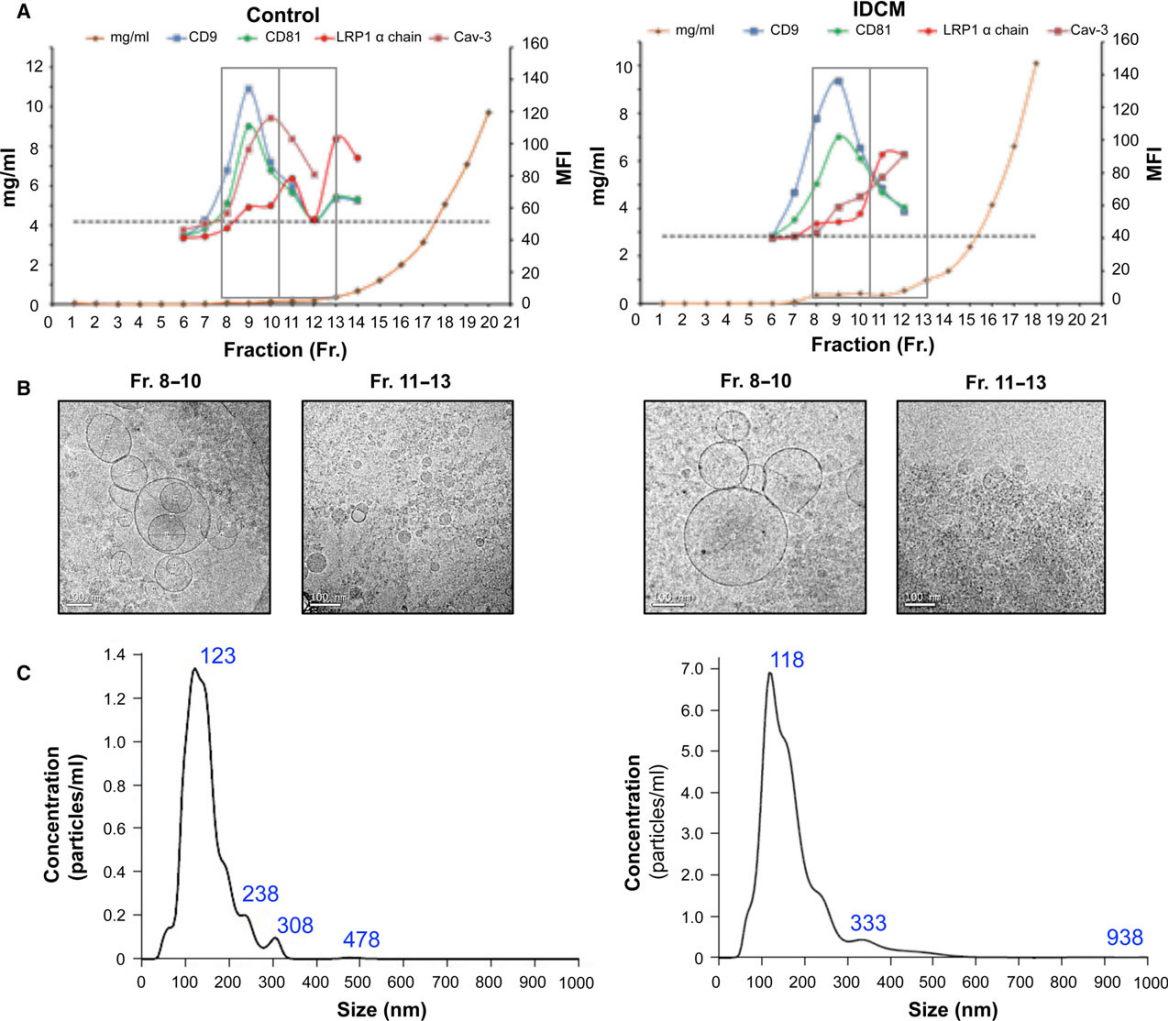
sLRP1 is not found in platelet‐free plasma‐derived extracellular vesicles (EV) fractions. (**A**) Representative analyses of platelet‐free plasma‐derived size‐exclusion chromatography (SEC) fractions by flow cytometry. Median fluorescence intensity (MFI) was measured, and the isotype negative control is represented as a dashed line. (**B**) Electron micrographs of vesicular protein‐enriched (8–10) and non‐enriched (11–13) fractions from a healthy control (left) and a idiopathic dilated cardiomyopathy (IDCM) patient (right) showing multifaceted assembly of round electron‐lucent membrane vesicles and a large quantity of smaller electron‐dense structures lacking a membrane bilayer, respectively. (**C**) Characteristic particle size distribution and concentration by NTA (healthy control, left; IDCM, right). *n* = 5 each control and IDCM. Scale bars = 100 nm.

To examine whether sLRP1 is exported out of the myocardium through EVs, methodology based on SEC was applied to platelet‐free plasma samples from both groups. SEC fractions were assessed by flow cytometry for enrichment with the EV‐related proteins CD9 and CD81. In particular, no differences in the measured levels of these EV markers were found between groups (*P* = 0.43 and *P* = 0.97 for CD9 and CD81, respectively). The set of fractions with the highest MFI values for CD9 and CD81 were then separated from the bulk of plasma proteins. Remarkably, the myocardial protein caveolin‐3 was detected in these fractions, but not LRP1 α chain in both groups (*P* = 0.99) (Fig. 4A).

Further analysis of the EV‐enriched fractions comprised assessment of the particle size distribution/concentration by NTA of the morphology and structure and cryo‐transmission electron microscopy to analyse the size range. Examination of representative images taken of the distinct SEC fractions confirmed the presence of membranous vesicles ranging from 40 to 300 nm in the fractions positive for vesicular markers, whereas non‐EV‐enriched fractions had an abundance of small electron‐dense particles lacking a discernible membrane bilayer, which most likely represent lipoproteins (Fig. 4B) 31. We also recognized particles within the expected size range of EVs without differences between groups (Fig. 4C).

## Discussion

The results from the present study indicate for the first time that the measurement of peripheral sLRP1 is potentially valuable for IDCM. Furthermore, as first approach, sLRP1 seems to be released to the circulatory system independent of the exosome pathway.

The protease inhibitor protein α‐2 macroglobulin, which is highly increased in the myocardium of IDCM patients 24, was one of the first LRP1 ligands described 32. In IDCM, specific cardiac alterations are produced mainly by an adverse remodelling of the left ventricle 33, 34, 35, where the activity of extracellular matrix metalloproteinases (MMPs) is deeply altered 36, 37. Increased activation of MMPs and negligible levels of specific MMP inhibitors are closely associated with the disease 30, 33, 38, 39, 40. Furthermore, one of the key LRP1 molecular relays is the mitogen‐activated protein kinase/extracellular signal‐regulated kinase (ERK) pathway 41, 42, 43. Excessive ERK signalling has been reported in IDCM patients 24, 44 and in experimental models of the disease 45. Data from our laboratory have also shown the redistribution and over‐activation of LRP1 within the localized, cholesterol‐ and glycosphingolipid‐enriched regions within plasma membranes, termed lipid rafts, in myocardium from IDCM patients, as well as a concomitant increase in non‐raft‐related ERK1,2/MMP9 24. Regarding myocardial levels of LRP1, although no statistical differences between IDCM and control hearts were previously found in our laboratory using a specific anti‐LRP1 β chain antibody 24, we here reveal that expression of LRP1 α chain is significantly promoted in IDCM. Moreover, whereas LRP1 β chain seems to be agglutinated in cardiac myocytes indistinctly in patients and controls, LRP1 α chain is mostly found in myocardial vessels in IDCM. These findings point out to complex cell‐specific regulatory mechanisms for the expression (and maybe assembly and cleavage) of the subunits conforming the LRP1 receptor.

The levels of circulating sLRP1 are significantly higher in patients with severe hypercholesterolaemia than in patients with moderate hypercholesterolaemia and normocholesterolemic controls 14. The association between circulating sLRP1 levels and pro‐atherogenic lipoproteins, including LDL cholesterol, is reproducible in different hypercholesterolaemic populations, suggesting their causality. High‐cholesterol diet and cholesterol‐lowering strategies also modulate inversely both vascular LRP1 expression and lipid content in the arterial intima 46, and atheroprotective strategies exert benefits related to increased communication between endothelial and smooth muscle cells *via* EVs 47. In the present study, however, we did not find a significant association between blood lipids and sLRP1 levels. A comprehensible explanation for this lack of association is that our patients cohort showed lower levels of LDL cholesterol to those in which this effect has been described (*i.e*. >190 mg/dl) 14. Furthermore, our preliminary analysis revealed no correlation between sLRP1 concentrations and treatment with statins. Nevertheless, additional work is warranted to evaluate the effects of atheroprotective strategies in the release and specific content of EVs carrying endothelial cell markers.

On the other hand, we have recently identified circulating sLRP1 as a novel biomarker of epicardial adipose tissue 21. Nevertheless, patients with congestive heart failure show significantly reduced amounts of epicardial adipose tissue compared to healthy individuals irrespective of the underlying aetiology of the cardiomyopathy 48, 49. Thus, despite epicardial adipose tissue was not quantitative monitored in our cohorts, it seems that this parameter would not play a role in determining circulating sLRP1 in this condition. At the histological level, a precise examination of myocardium afflicted by IDCM confirmed the characteristic hallmarks, such as altered cardiac muscle integrity, myocyte atrophy, and increased deposition of collagen and lipids around myocardial filaments 50. We also showed that the TG content is higher in myocardium from IDCM patients than control subjects. In contrast, myocardial CE levels were extremely low and similar to controls. Although some pharmacological treatments might influence these findings, they may be related to a range of well‐reported metabolic dysfunctions, including altered fatty acid uptake and oxidation in failing hearts 51, 52. Thus, extra‐ or intra‐myocardial cholesterol does not seem to be related to higher sLRP1 release to the plasma of IDCM patients. Previously, plasma sLRP1 was shown to antagonize ligand endocytosis by cellular LRP1 in the lungs of patients with acute respiratory distress syndrome 19, preventing the cellular clearance of MMPs. A similar mechanism may contribute to tissue remodelling in IDCM. Thus, we sought to determine whether LRP1 cleavage and secretion is promoted in the circulatory system in IDCM patients. We found that circulating levels of sLRP1 are significantly increased in patients with IDCM, but future investigations are warranted to elucidate whether LRP1 shedding is promoted or sLRP1 clearance is decreased in this condition.

In the present study, we also assessed whether LRP1 is exported out of the myocardium *via* the EV pathway. EVs include a wide range of lipid membrane vesicles secreted by all types of cells, distinctive in size, biogenesis, cargo molecules and function 53. After being secreted upon the fusion of multivesicular bodies with the plasma membrane, EVs play central roles in a variety of processes, including intercellular communication, recycling of membrane proteins and lipids, immunomodulation, senescence, angiogenesis, proliferation, differentiation and migration. Interestingly, shed under both normal and pathological conditions, EVs have been found in different biological fluids (*e.g*. blood, urine, saliva) and are considered relevant diagnostic tools and a key source of biomarkers 54. Thus, the role of EVs in cardio‐metabolic diseases has been widely studied in the last few years 55. To our best knowledge, this is the first study to assess the presence of sLRP1 in plasma‐derived EVs extracted by SEC technology. Although LRP1 is preferentially detected at the intracellular level using detergent‐based analytical techniques such as Western blot or immunocytochemistry, some previous studies have measured its cellular expression levels in standardized flow cytometry assays 56, 57. Using SEC, protein content was undetectable in SEC‐pooled EV fractions (fractions 8‐10), as most of the soluble proteins eluted in later fractions (fraction 13 onwards). This finding confirms SEC as the cleanest method to isolate EV‐enriched samples removing most of the over‐represented plasma proteins for subsequent sophisticated analysis such as proteomics 30. Indeed, our analyses suggested that LRP1 is not associated with this type of circulating EVs and that these EVs seem to contain the specific myocardial marker caveolin‐3 24. Currently, together with the fact that we cannot assume that these peripheral EVs are entirely exported out from myocardium, this finding counteracts the possibility of tracking the exact origin of the sLRP1 found in circulation. Thus, we cannot exclude the possibility that sLRP1 is released from other body sources. To that end, future studies and funding priorities should include the analysis of EVs comprised in blood samples from the coronary sinus and how sLRP1 elutes later in non‐EV fractions (fractions 11‐13), when protein amount is extremely low. Although we cannot exclude some association with carrier proteins and/or small lipoprotein particles, we also speculate that this is caused in part by the high molecular weight of LRP1 α chain (~515 kDa).

As a limitation, our study included blood samples collected from ambulatory patients and myocardial tissue specimens extracted from patients undergoing heart transplantation. Thus, analysis of specimens collected from the same group of patients, preferably in those undergoing heart transplantation, should be recommended to further confirm the clinical value of sLRP1 measurement. Nevertheless, our study opens new doors for further comprehensive analysis of the cargo of these EVs release into the systemic blood of IDCM patients. Additional studies with a higher number of EV samples for each group should be performed to corroborate these preliminary results. For instance, proteomics analysis could support our findings and provide additional information on the molecular basis of the disease and assist in the search for novel blood markers with robust diagnostic and prognostic value 25.

In summary, our results support that endoproteolysis and secretion of LRP1 is promoted in IDCM and that circulating levels of its soluble form may be a potentially helpful measurement. Moreover, sLRP1 appeared to be secreted independently of EVs, a fact that does not favour a precise identification of its origin. In line, specific *in vivo* models of disease (*e.g*. pacing‐induced heart failure animal models) are needed to establish the exact mechanism and provenance of sLRP1 release. As mentioned previously, it will be crucial to validate a versatile clinical utility for the measurement of sLRP1 in larger cohorts of patients with IDCM. To that end, large, well‐designed, multi‐centre, collaborative studies are central to showing that it can be a highly sensitive and cost‐effective measurement with reliable clinical value. Further insights into the potential associations between sLRP1 concentrations, levels of EVs in circulation, cardio‐metabolic diseases, epicardial adipose tissue, clinical variables and/or events, cardiac function parameters or degree of tissue damage are also mandatory. We also highlight the need for continual development of analytical techniques to permit quantitative comparisons of EV levels in circulation, as new data are collected. As the number of functional studies on EVs increases, innovative workflow technical platforms are necessary to specifically characterize surface and cargo proteome. In this way, it is crucial to determine the detailed orientation and topology of proteins (*e.g*. LRP1 and their proteolytically cleaved forms) on or inside of EVs before they can reach clinical significance as biomarkers for disease. Particularly, in IDCM, biomarker research is in the early development stage and the usefulness of EVs and the recognition of their specific combinations of proteins, lipids and miRNAs as possibly helpful clinical tools has enormous potential.

## Funding source

This work was supported by grants from the Ministerio de Educación y Ciencia (SAF2014‐59892‐R), the Ministerio de Economía y Competitividad (Juan de la Cierva, JCI‐2012‐14025), Fundació La MARATÓ de TV3 (201502, 201516, 201521_10), the Fundació Daniel Bravo Andreu, the Sociedad Española de Cardiología, the Societat Catalana de Cardiologia, the Generalitat de Catalunya (SGR 2014, CERCA Programme), and the Fundació Bancària La Caixa. This work was also funded by the Red de Terapia Celular – TerCel (RD16/0011/0006), the CIBER Cardiovascular – (CB16/11/00403) and Fondo de Investigación Sanitaria, Instituto de Salud Carlos III (FIS PI14/01682, FIS PI14/01729 and CD14/00109) as part of the Plan Nacional de I+D+I cofounded by ISCIII‐Sudirección General de Evaluación y el Fondo Europeo de Desarrollo Regional (FEDER).

## Conflicts of interest

The authors have no potential conflict of interests to declare.

## Supporting information


**Fig. S1** Altered collagen deposition in IDCM myocardium.

**Appendix S1** Methods.
