# Supplementary material for: Extracellular vesicles do not contribute to higher circulating levels of soluble LRP1 in idiopathic dilated cardiomyopathy
Source: J Cell Mol Med. 2017 May 29;21(11):3000–9. doi: 10.1111/jcmm.13211 (PMC5661250; doi:10.1111/jcmm.13211)
Supplement: Supplementary file 2 — Appendix S1 Methods. [file JCMM-21-3000-s002.docx]

**Supplementary Methods**

**Extracellular Vesicle Isolation and Characterization**

The isolation of extracellular vesicles (EVs) by size-exclusion chromatography was performed using a 12 mL of Sepharose CL-2B (Sigma Aldrich), which was stacked in a 20-mL syringe (BD Plasticpack^TM^), and washed and equilibrated with 1X phosphate buffered saline (PBS) (GIBCO^®^ Life Technologies). Different columns were used for each sample with the pertinent washing steps between different samples. Up to 2 mL of serum was loaded onto the column and a total of 20 fractions collected (0.5 mL per fraction) using 1X PBS as elution buffer.

Subsequently, the size distribution and concentration of plasma-derived EVs were assessed in a NanoSight LM10 instrument (Malvern Instruments Ltd) equipped with a 638 nm laser and a CCD camera (model F-033). The data were then analyzed by the nanoparticle tracking analysis (NTA) software (versions 3.1 build 3.1.46). The detection threshold was set to 5, and the blur and max jump distance were set to auto. Samples were diluted 90-100 times with sterile and filtered 1X PBS to reduce the number of particles in the field of view to <140/frame. Readings were taken in single capture mode over 60 s at 30 frames/s at a camera level set to 16, and manual temperature monitoring.

In addition, 50 μL of each size-exclusion chromatography-purified fraction were analyzed for the presence of specific vesicular markers (CD9 and CD81), caveolin-3, and LRP1 α chain by flow cytometry as described previously. Succinctly, they were incubated with aldehyde/sulphate-latex beads (4 μm; Invitrogen) for 15 min at room temperature. 1X PBS supplemented with 0.1% BSA and 0.01% NaN_3_ was used as bead-coupling buffer (BCB) and the mix incubated overnight at room temperature on continuous rotation. Centrifugation was carried out at 2,000 x g for 10 min to spin down the EV-coated beads, followed by another washing step with BCB and a second centrifugation at 2,000 x g for 10 min. The EV-coated beads were then labeled at 4ºC for 30 min with 5 μl of the following antibodies: anti-CD9 (Clone VJ1/20), anti-CD81 (Clone #G0709; Santa Cruz Biotech), anti-caveolin-3, and anti-LRP1 α chain (BD Pharmingen). Polyclonal isotype antibodies (Abcam) were used as negative controls. The antibody excess was eliminated with another BCB washing step. EV-coated beads were then incubated with FITC-conjugated secondary antibodies (Southern Biotech) for 30 min, washed twice with BCB, and analyzed using a FacsVerse flow cytometer (BD Biosciences). Mean fluorescence intensity and FlowJo software (Tree Star) were used to compare the resulting fractions.

**Myocardial Lipid Content Analysis**

After homogenization of myocardial tissue in NaOH 0.1 M, its lipid content including cholesteryl sters (CE), free cholesterol (FC) and triglyceride (TG) was analyzed by thin layer chromatography. To that end, the organic solvent was removed under an N_2_ stream, the lipid extract redissolved in dichloromethane, and one aliquot (100 μL) partitioned on silica G-24 plates. Different concentrations of standards (a mixture of cholesterol, cholesterol palmitate, and TGs) were applied to each plate. The chromatographic developing solution was heptane/diethylether/acetic acid (74:21:4, vol/vol/vol). Spots corresponding to CE, TG, and FC were quantified by densitometry against the standard curve of cholesterol palmitate, TGs, and cholesterol, respectively, using a computing densitometer (Molecular Dynamics, Inc.).
