# Supplementary material for: Extracellular vesicles do not contribute to higher circulating levels of soluble LRP1 in idiopathic dilated cardiomyopathy
Source: J Cell Mol Med. 2017 May 29;21(11):3000–9. doi: 10.1111/jcmm.13211 (PMC5661250; doi:10.1111/jcmm.13211)
Supplement: Supplementary file 3 [file JCMM-21-3000-s003.docx]

**Supplementary Figures**

**Supplementary Figure 1. Altered collagen deposition in IDCM myocardium**. Representative bright-field microscope images of LV samples stained with Sirius red distinguishing total Col (red) and cardiac muscle (yellow). Polarized light images showing Col I (red/yellow) and Col III (green) fibrils distributed within the same sections are also shown. n = 5 each control and IDCM. Scale bars = 20 μm.
